# The impact of stroke on intramuscular connective tissue in mice

**DOI:** 10.3389/fneur.2026.1824344

**Published:** 2026-08-13

**Authors:** Xiaoxiao Zhao, Giulio Morri, Matteo D’Urso, Caterina Fede, Lucia Petrelli, Livia Vignozzi, Yunfeng Sun, Claudia Clair, Gabriele Deidda, Preeti Raghavan, Manuela Allegra, Carla Stecco

**Affiliations:** 1 Department of Neurosciences, Institute of Human Anatomy, University of Padova, Padova, Italy; 2 Padova Neuroscience Center (PNC), University of Padova, Padova, Italy; 3 Neuroscience Institute, National Research Council (CNR), Padova, Italy; 4 Department of Biomedical Sciences, University of Padova, Padova, Italy; 5 Faculty of Medicine and Dentistry, Queen Mary University of London Malta Campus, Victoria, Malta; 6 Department of Physical Medicine and Rehabilitation, Department of Neurology, Johns Hopkins University School of Medicine, Baltimore, MD, United States

**Keywords:** collagen, extracellular matrix, hyaluronan, intramuscular connective tissue, stroke

## Abstract

**Background:**

Previous studies about post-stroke peripheral structural remodeling have been focused on muscle fiber alterations, while extracellular matrix (ECM) remodeling in musculoskeletal tissues remains unclear. This study examines changes in extracellular matrix (ECM), specifically hyaluronan (HA) and collagen, in forelimb muscles following distal middle cerebral artery occlusion (dMCAO) in mice.

**Methods:**

Twenty-two 8-week-old C57BL/6 mice (10 male, 12 female) were randomly assigned to the control group (*n* = 10; 4 male, 6 female) or the experimental group (*n* = 12; 6 male, 6 female). 4 weeks post-stroke, forelimb muscle samples were collected and analyzed for HA and collagen distribution and concentrations.

**Results:**

HA concentrations were significantly elevated in both the left (stroke affected) forelimb muscles (63.74 ± 11.80 μg/g), and right (unaffected) forelimb muscles (64.16 ± 13.52 μg/g) of stroke mice compared to the controls (left: 50.07 ± 8.84 μg/g, *p* = 0.022; and right: 49.36 ± 12.22 μg/g, *p* = 0.015). No significant difference in HA levels was observed between the affected and unaffected sides of stroke mice (*p* = 0.905). Collagen concentrations did not differ significantly among groups (control left: 8.62 ± 2.91 μg/g; control right: 8.77 ± 1.35 μg/g; stroke-affected: 8.24 ± 2.11 μg/g; stroke-unaffected: 8.28 ± 2.45 μg/g).

**Conclusion:**

The results revealed a systemic increase in HA concentration in the stroke group, while collagen levels remained unchanged. These findings provide biochemical evidence of early alterations in extracellular matrix composition after stroke.

## 1 Introduction

Stroke is a major cause of long-term disability worldwide (1), with post stroke motor impairment affecting about 80% of stroke survivors involving the face, upper and lower limbs (2). More than a half of all stroke survivors experience upper limb dysfunction, and around 50% of them suffer a continuous dysfunction 6 months post stroke during the chronic stage (3, 4). Previous research about post-stoke recovery has been focused on central nervous system (CNS) plasticity (e.g., cortical reorganization, axonal sprouting) (5–8) and non-neurogenic changes involving muscle fiber elements adaptations (e.g., atrophy, fiber-type switching) (9–12). However, these mechanisms are unable to fully explain the chronic musculoskeletal complications observed in stroke survivors, such as spasticity, reduced range of motion, and altered tissue mechanics.

Recent evidence suggests that altered tissue mechanics such as stiffness and viscosity following stroke contribute to the development of spasticity and the increase in muscle and joint stiffness, particularly in the chronic stages of stroke (13–15). In stroke survivors with spasticity, two studies reported significantly increased muscle stiffness, attributed to structural alterations in muscle fiber and intramuscular connective tissue (IMCT), leading to reduced range of motion (14, 15). Chardon et al. (13) further identified a 40–57% increase in musculotendon stiffness on stroke affected side, likely reflecting ECM fibrosis. Findings of viscosity are inconsistent. Lindberg et al. (16) observed lower viscosity in stroke patients while Wang et al. (15) found increased viscosity in stroke group which correlated with spasticity severity. Despite this difference, both studies proposed that changes in viscosity may be attributed to extracellular matrix remodeling, which can exacerbate spasticity severity (17). These findings emphasize the importance of ECM alterations in IMCT in post-stroke dysfunction.

Skeletal muscle is composed of muscle fibers and intramuscular connective tissue (IMCT), which refers to endomysium, perimysium, and epimysium (18), which transmit force, regulate muscle flexibility and stiffness through its ECM components (19). The ECM consist of protein fibers and ground substance (19, 20). Collagen, the most abundant protein fibers in the ECM, provides structural support for muscle fibers and play a key role in force transmission (21). Elevated collagen levels have been shown to increase the stiffness of the IMCT and the whole muscle (22). The main component of the ground substance is Hyaluronan (HA) (23). It is a high molecular weight glycosaminoglycan (GAG), playing a crucial role in facilitating the gliding between IMCT and muscles (24). Changes in HA amount or molecular weight can alter tissue viscosity and the ability to facilitate tissue gliding (25). Altered viscosity is associated with impaired IMCT gliding, which can increase tissue stiffness and contribute to myofascial pain (24). Although previous studies have attributed that muscle mechanical alterations in chronic stroke stage to ECM changes, direct biochemical quantification of these ECM alterations in muscle tissue in chronic stroke stage remains limited.

To fill this gap, we quantified ECM changes, specifically HA and collagen concentrations, in forelimb muscles following MCAO stroke in mice, to provide biochemical evidence to ECM targeted interventions for post-stroke rehabilitation.

## 2 Methods

### 2.1 Animals

All procedures were performed according to the guidelines of the Italian Ministry of Health for care and maintenance of laboratory animals, and in strict compliance with the European Community Directive n. 2010/63/EU on the protection of animals used for scientific purposes. Animal experimentation at the University of Padova was approved by the Italian Ministry of Health (nr 935/2024-PR). Mice were housed in standard condition cages with a 12 h light/dark cycle and received food and water ad libitum. A total of twenty-two, 8-week-old, C57BL6J mice were used in this study. Young adult mice were intentionally selected to avoid age-related extracellular matrix alterations and to ensure that the observed changes in hyaluronan and collagen were primarily attributable to stroke-induced effects. Animals were randomly allocated into two groups: the stroke (*n* = 12) and the sham control (*n* = 10). And the experimenter performing behavioral, histological and biochemical analyses remained blinded to group allocation until completion of data analysis.

### 2.2 Middle cerebral artery occlusion surgery

Mice were anesthetized with isoflurane (4% for induction, 1.5–2% for maintenance) mixed with O_2_. Combined analgesia, Dexamethasone (2 mg/kg, Dexadreson, MSD Animal Health, Italy) and Caprofen (5 mg/kg, Rimadyl, Zoetis, Italy), was administered through intramuscular and intraperitoneal injections, respectively, before any surgical intervention. During the surgical procedure, body temperature was continuously maintained at ~37 °C with a heating pad (KF Technology, Italy). Distal Middle Cerebral Artery Occlusion (dMCAO) was performed as documented by Llovera and colleagues (26). Briefly, on the right the region between the ear and the eye was shaved and disinfected with an iodine-povidone solution. A 2.5% lidocaine cream was applied to the target area to secure local analgesia. For the experimental group, a 4 mm horizontal incision was made between the eye and the ear canal, followed by a 4–5 mm vertical incision extending from the ear canal. This allowed the creation of a skin flap that could be retracted. Identical incisions were then made in the temporalis muscle to expose the underlying temporal bone. The right MCA was identified beneath the skull, and the bone above the artery was thinned using a drill until translucent and then carefully removed with forceps. The right MCA was eletrocoagulated under the bifurcation with 0.25 mm bipolar forceps (GIMA, Italy) connected to an electrosurgical unit set to 7 W (Diatermo 122, GIMA, Italy). After confirming no blood flow via gentle touch, the temporalis muscle and the skin were repositioned and glued with veterinary glue (Vetbond, 3 M Animal Care Products, St. Paul, MN, United States). For the control group, the sham operation procedure involved partially detaching temporal muscle to create a muscle flap while keeping the muscle intact. Then the incision was closed. After surgery, the animals were placed in a recovery box at 32 °C until fully awake.

### 2.3 Behavioral characterization of motor function

The motor function was evaluated by the gridwalk test at different time points, before (baseline) and after surgery (day D30). Briefly, as described previously (26), the animals were placed on an elevated grid with evenly spaced square openings (11 mm per side) and were allowed to explore freely while their movements were video recorded for subsequent analysis. The test captured spontaneous motor behavior and detected foot placement errors (foot faults). The total number of steps (with left and right paws) and foot faults (of the left impaired paw) were counted. The Foot Fault Percentage (FFP) is calculated using the formula:


$$\mathrm{FFP}=\frac{\text{Left Foot Faults}}{\text{Total Steps}}\times 100\%$$

Higher percentages indicate greater motor impairment, while lower percentages suggest better motor coordination and recovery. This test provides a sensitive measure of motor impairment in models of neurological injury, such as stroke, and does not require pre-test training or external motivation, making it a straightforward and effective tool for assessing motor function recovery.

### 2.4 Sample collection

**Figure 1:**
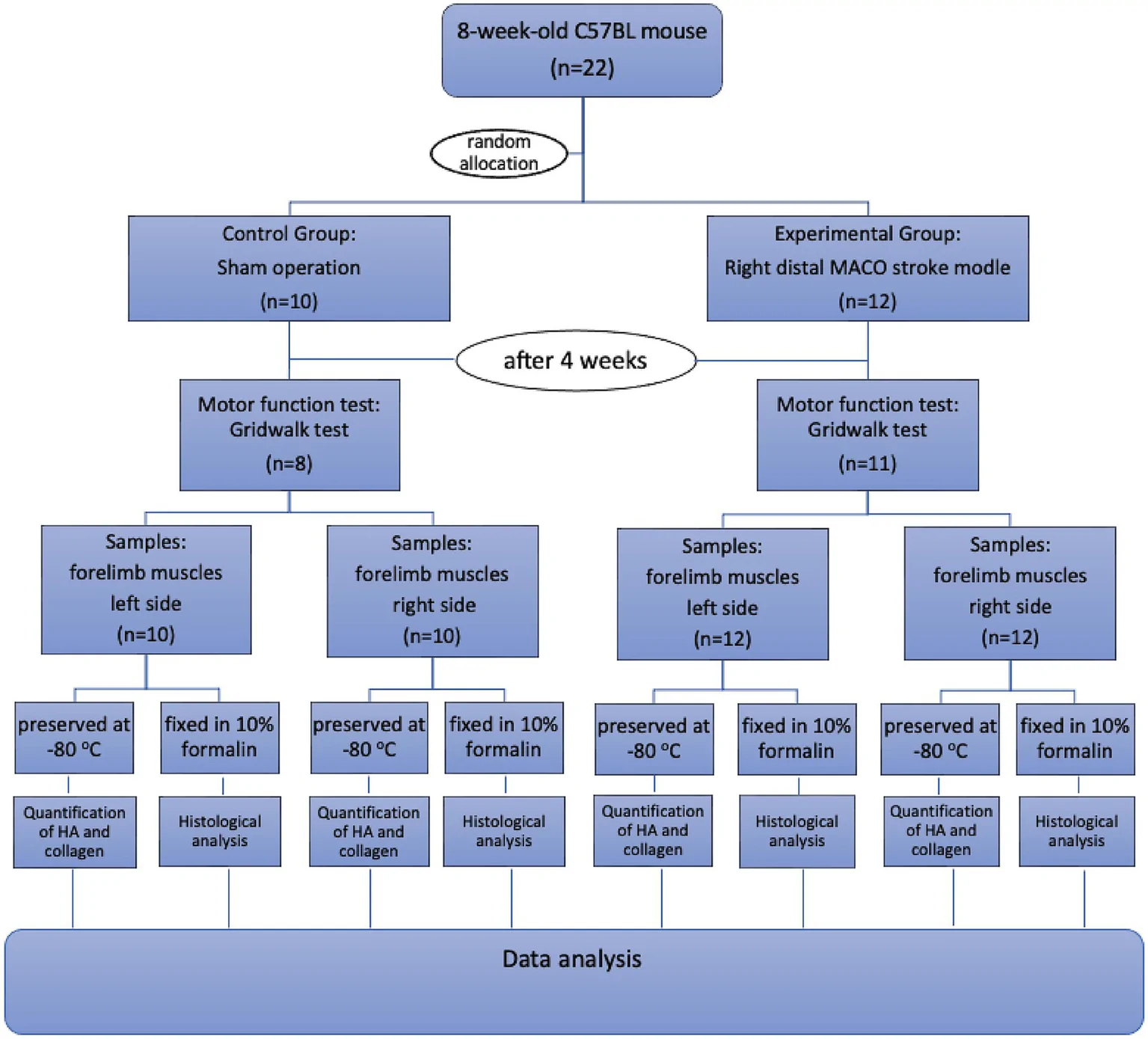
Experimental design: Schematic overview of the experimental workflow. Eight-week-old C57BL/6 J mice were randomly assigned to a sham-operated control group or to a right distal middle cerebral artery occlusion (dMCAO) group. Motor function was assessed using the gridwalk test 4 weeks after surgery. Left and right forelimb muscles were collected for biochemical quantification of hyaluronan and collagen and for histological staining.

In line with previous stroke studies, the 4-week post-stroke time point was selected to represent the chronic stage (27–29). Four weeks post-surgery (at D30), animals were deeply anaesthetized with urethane (16.5%, 1.65 g/kg, Sigma Aldricht) and then transcardially perfused with 20–30 mL PBS, followed by paraformaldehyde (PFA) diluted at 4% in 0.1 m phosphate buffer, pH 7.4. After brains were collected, then the left and right forelimb were excised from the mouse in the experimental and the control group. After the skin of forelimb and the paws was removed, the entire distal part of the fore limb including muscles (extensor carpi radialis, extensor carpi ulnari; flexor carpi ulnaris; flexor digitorum profundus) and bones (radius and ulna) was fixed in 10% formalin before decalcification and the histological and immunohistochemical analysis. For the proximal part of the forelimb, the muscle samples (including biceps brachii and triceps brachii) were excised from humerus of the mouse and preserved at −80 °C for the HA and collagen assay. The procedures are demonstrated in Figure 1.

### 2.5 Brain histological confirmation of the dMCAO model

Following transcardial perfusion, brains were collected and post-fixed in 4% paraformaldehyde. Coronal brain sections (60 μm thick) were cut using a microtome (Leica SM2010R), and a one-in-six series of sections was stained with Hoechst. Representative sections were then used to confirm successful induction of cortical infarction following dMCAO and to visualize the rostrocaudal extent of the core lesion by epifluorescence microscopy.

### 2.6 Quantification of hyaluronan

The Purple-Jelley HA assay (Biocolor Ltd. Carrickfergus, United Kingdom) was utilized to measure the concentration of HA in forelimb skeletal muscles of the mouse. Following previously established protocols (30–32), 150 mg ± 50 mg of wet tissue (defrosted from −80 °C) was weighted, finely chopped by a surgical scalpel, and transferred into 2.0 mL microcentrifuge tubes. Then the samples were digested overnight at 55 °C in 400 μL of 50 mM TRIS–HCl (pH 7.6) containing 20 μL Proteinase K (Sigma) to ensure complete enzymatic digestion. After digestion, the samples were centrifuged at 13000g for 10 min. Then the supernatants were transferred to new microcentrifuge tubes and mixed with 1.0 mL of a GAG precipitation reagent. Following a 15-min incubation in the GAG precipitation reagent at room temperature, the samples underwent another centrifugation at 13000g for 10 min, producing GAG-rich precipitate (pellet) that were subsequently resuspended in water and mixed with 2 M NaCl and cetylpyridinium chloride (CPC). After repeating the afore mentioned steps and successfully recovering the total GAG content, HA was isolated by adding 500 μL of 98% ethanol to the samples, followed by centrifugation and full hydration in 100 μL of water. For colorimetric analysis, 200 μL of a purple dye reagent supplied with the Purple-Jelley HA assay kit (Biocolor Ltd., Carrickfergus, United Kingdom) was added to 20 μL aliquots of the test samples, standards, and reagent blanks.

Absorbance was measured at 650 nm using a Wallac Victor3 1420 Multilabel Counter (Perkin Elmer, Finland) in 96-microwell plates. The HA concentrations were determined by comparing the absorbance values to a standard curve created with an HA standard (200 μg/mL). Finally, the HA content in the original tissue samples was calculated by converting the absorbance values into micrograms of HA per 100 μL, followed by determining the average micrograms of HA per gram of wet tissue for each sample.

### 2.7 Quantification of collagen

The Total Collagen Assay Kit (Abcam Ltd., Cambridge, United Kingdom) was used to measure collagen concentration in the forelimb skeletal muscle of the mouse. As described previously (32, 33), 60–100 mg of wet tissue (defrosted from −80 °C) was weighed and finely chopped into small pieces by a surgical scalpel. Then the fragments were mechanically homogenized in distilled water at a ratio of 100 μL per 10 mg of tissue. For each sample, 100 μL of the homogenate was combined with 100 μL of 10 N NaOH and heated at 120 °C for 1 h to extract collagen. After cooling on ice and neutralizing with 100 μL of 10 N HCl, the samples were centrifuged at 10000g for 5 min. From the supernatant, 10 μL was transferred to a 96-microwell plate, which was then placed in an oven at 65 °C for 10–20 min until hydroxyproline (white crystals) formed. Next, 100 μL of oxidizing reagent (prepared by mixing 6 μL Chloramine T with 94 μL oxidation buffer) was added to each well and incubated at room temperature for 20 min. For color development, 50 μL of developer solution was added and incubated at 37 °C for 5 min, followed by the addition of 50 μL of concentrated dimethylaminobenzaldehyde (DMAB). The plate was thoroughly mixed and incubated on a plate heater at 65 °C for 45 min.

After incubation, absorbance was measured at 570 nm using the Wallac Victor3 1420 Multilabel Counter (Perkin Elmer, Finland). The results were compared against a standard curve generated from Collagen I standard solution (ranging from 0 to 18 μg/mL) to calculate collagen content in micrograms per milligram of tissue.

### 2.8 Decalcification for the forelimb of mouse

Decalcification was carried out using 10% ethylenediaminetetraacetic acid (EDTA) following a standardized protocol. First, the fixed forelimbs were incubated in 10% formalin for 24 h, then washed in running water for 20 min and then rinsed twice in PBS (20 min each). The samples were subsequently immersed in 10% EDTA, with the solution replaced weekly for 2 weeks. Finally, the forelimbs were washed twice in running water (20 min each) and rinsed twice in distilled water before being prepared for paraffin embedding, sectioning, and subsequent histological and immuno-histochemical staining.

### 2.9 Histological analysis

Formalin-fixed samples (forelimb muscle with radius and ulna) were dehydrated in graded ethanol and xylene, before being embedded in paraffin. Sections of 5 μm thickness were cut by a microtome, then dewaxed and subjected to Picrosirius Red staining for collagen and immunohistochemistry staining for HA distribution.

Picrosirius Red staining: Dewaxed tissue sections were stained with Picrosirius Red solution for 15 min to specifically highlight collagen fibers. Then the sections were quickly rinsed in 0.01 M hydrochloric acid (HCl) for 20 s and washed in deionized water. After dehydration through a graded alcohol series and clearing in xylene, the samples were mounted using Eukitt (Agar Scientific) (34).

Immunohistochemistry: Dewaxed tissue sections were treated with 0.5% H_2_O_2_ for 15 min to block endogenous peroxidase activity. Following thorough washing in phosphate-buffered saline (PBS), the specimens were incubated in a 0.2% blocking solution consisting of 0.2% Bovine Serum Albumin (BSA) in PBS for 1 h, then incubated in anti-HABP (Hyaluronic Acid Binding Protein; dilution 1:1000, Millipore Sigma-Aldrich, MI, Italy) overnight at 4 °C. Following PBS washes, the samples were incubated with HRP-conjugated Streptavidin (Jackson ImmunoResearch, Cambridgeshire, United Kingdom; dilution 1:250) for 30 min, then washed three times with PBS. The reaction was developed using 3,3′-diaminobenzidine (Liquid DAB + Substrate Chromogen System Kit, Dako, CA, United States) and stopped using distilled water. Finally, the slides were dehydrated and mounted with Eukitt (Agar Scientific).

### 2.10 Image acquirement

All the images were acquired using the Leica DMR microscope (Leica Microsystems, Wetzlar, Germany) at 10× magnification.

### 2.11 Statistical analysis

Statistical analyses were performed using IBM SPSS software (version 25, SPSS, Chicago, Illinois, United States), with a significance level set at *p* < 0.05. Normality was assessed by the Shapiro–Wilk test, while homogeneity of variance was evaluated by Levene’s test. Behavioral test (gridwalk test) results are presented as mean ± standard error of the mean (SEM). Biochemical measurements (HA and collagen concentration) results were expressed as mean ± standard deviation (SD). For behavioral data, paired *t*-tests were applied to compare baseline and day 30 values within the same animals, while unpaired (independent) *t*-tests were used to compare control (sham surgery) and stroke groups at day 30. For biochemical data, comparisons of HA and collagen concentration in the forelimb muscle among the left (stroke affected), right (unaffected), and control (sham surgery) groups were conducted using one-way analysis of variance (ANOVA) test. Dunnett’s *post hoc* test was applied to compare the HA and collagen concentration in the forelimb muscle of the left (stroke affected) and right (unaffected) sides in the experimental group separately with those of the left and right sides in the control group. In addition, paired *t*-test were used to compare the HA and collagen concentrations between the left and right forelimb muscles within the same animals in the experimental sham group.

## 3 Results

### 3.1 Ischemic stroke induces fine sensorimotor impairments in adult mice

**Figure 2:**
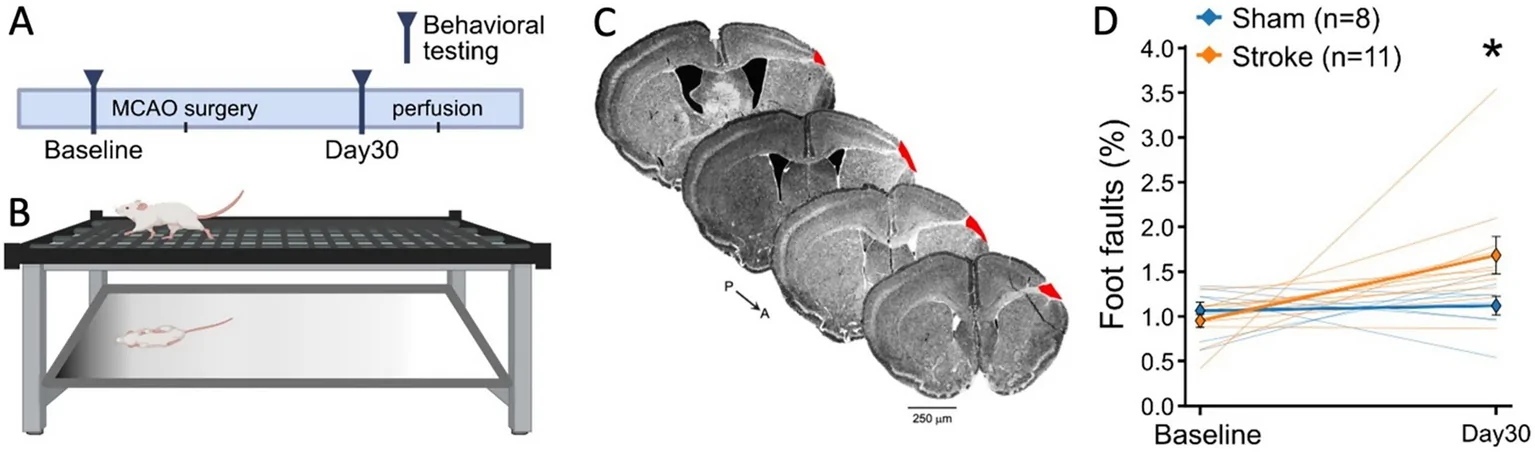
dMCAO induces fine sensorimotor impairments in adult mice. **(A)** Experimental timeline. **(B)** Cartoon showing the experimental setup of the gridwalk test. **(C)** Representative serial coronal brain sections (60 μm thick), stained with Hoechst, of an example stroke mouse, illustrating the extent of the ischemic lesion. The lesion core is highlighted in red. **(D)** Quantification of the foot faults (expressed as percentage of the baseline of each animal) at baseline and 30 days after surgery, for sham (blue, *n* = 8) and stroke (orange, *n* = 11) animals. Color-coded curves represent single sham (light blue) and stroke (light orange) animals. Data are presented as the mean ± SEM. *p < 0.05.

To explore whether an ischemic stroke could affect sensorimotor function, thus inducing changes in the muscular composition of the impaired forelimb, we used the MCAO model and assessed the sensorimotor function (Figure 2A). Ischemic stroke was induced in adult mice by performing the occlusion of the MCA, which produced a core lesion located in the somatosensory cortices, leaving the primary motor cortex perilesional to the core. Representative serial coronal brain sections stained with Hoechst confirmed successful induction of cortical infarction following dMCAO (Figure 2C). The lesion was located within the expected middle cerebral artery (MCA) territory and extended across multiple rostrocaudal levels. Given the primary motor cortex perilesional to the lesion, we used the gridwalk test to evaluate the behavioral outcome before (at baseline) and 30 days (D30) after stroke or sham surgery. Fine sensorimotor behavior was assessed by computing correct steps and foot-faults, namely, steps not providing body support with the foot falling into a grid hole (Figures 2B,D). After stroke, the sham control group exhibited stable performance (*n* = 8, mean ± SEM, 1.121 ± 0.105), similar to its baseline condition (1.066 ± 0.095; baseline versus D30, paired *t*-test *p* = 0.2665; Figure 2D), while the stroke animals displayed a long-lasting sustained impairment at D30 (*n* = 11; baseline, 0.952 ± 0.073; D30, 1.684 ± 0.209; paired *t*-test, *p* = 0.0038; sham versus stroke at D30, *t*-test, *p* = 0.0468; sham versus stroke at baseline, *t*-test, *p* = 0.3524; Figure 2D). These results indicate that the MCAO induces sensorimotor deficits in adult mice.

### 3.2 Quantification and distribution of HA in the forelimb muscles

**Figure 3:**
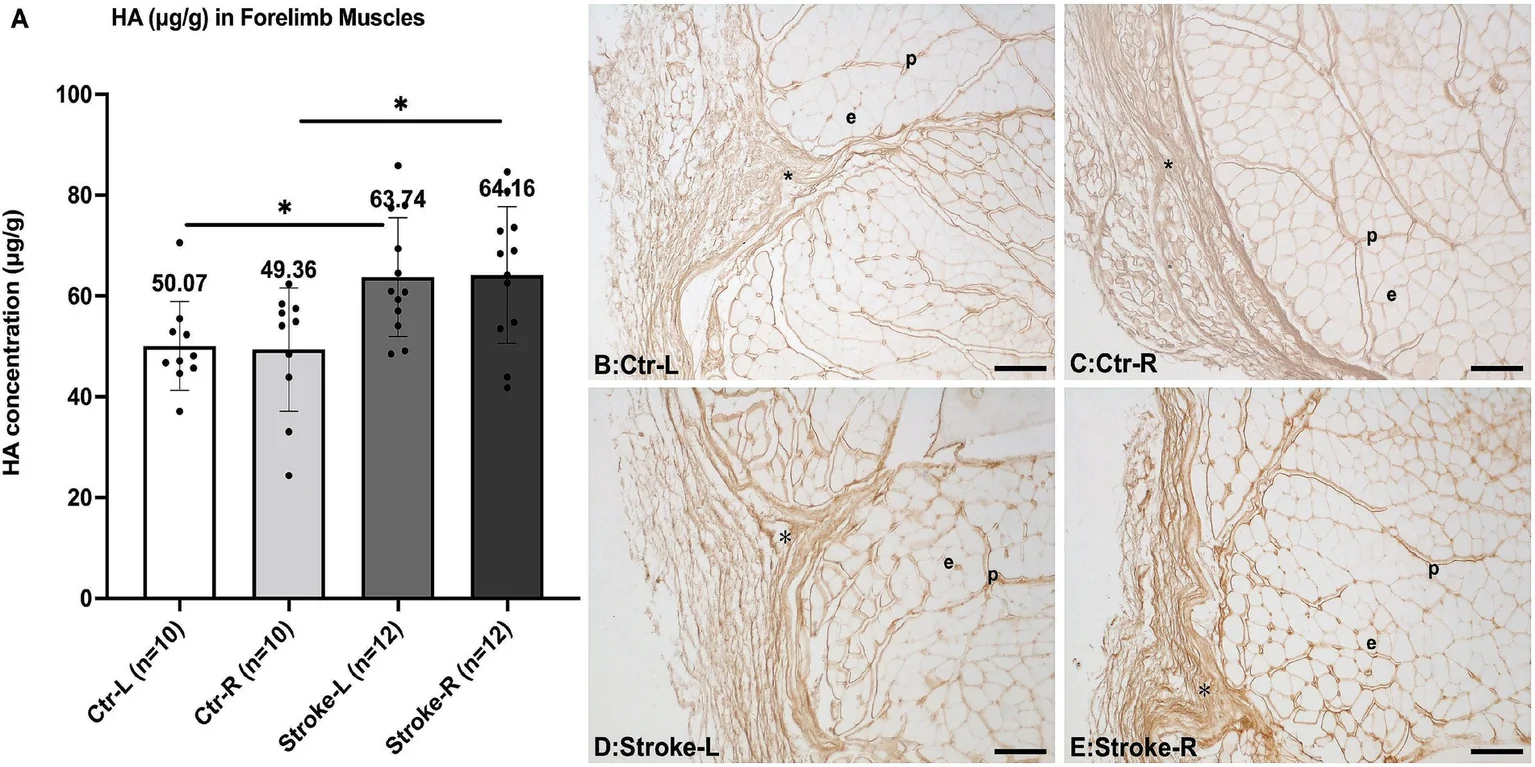
**(A)** HA concentration in forelimb muscles. The HA concentration in the forelimb muscles both on the left (Ctrl-L), the right (Ctrl-R) side of control mouse, and on the left (Stroke-L, stroke affected) side, the right (Stroke-R, non-affected) side of stroke mouse (Data are presented as the mean ± SD, *p < 0.05). Paired *t*-tests were applied to compare the HA concentrations between the left and right forelimb muscles within the same animals in the control and experimental group. ANOVA with Dunnett’s *post hoc* test was applied to compare the HA concentrations between the left and right sides of the experimental group with the left and right sides of the control group separately. **(B–E)** HA distribution in IMCT of the forelimb muscle. Anti-HABP (Hyaluronic Acid Binding Protein) staining; HA is in brown. **(B)** Control left side; **(C)** Control right side; **(D)** Stroke left (affected) side; **(E)** Stroke right (unaffected) side. *, Epimysium; p, Perimysium; e, Endomysium. Scale bars: 100 μm.

The HA concentrations in the forelimb muscles were: 50.07 ± 8.84 μg/g on the left and 49.36 ± 12.22 μg/g on the right side of the sham surgery mouse in the control group; 63.74 ± 11.80 μg/g on the left (stroke affected) side, 64.16 ± 13.52 μg/g on the right (non-affected) side of the stroke mouse in the experimental group (Figure 3A). Compared with controls, HA concentration was significantly higher in stroke affected mouse on both sides (left: *p* = 0.022, right: *p* = 0.015). Within group comparisons showed no left and right difference in either the control group (*t* (9) = 0.147, *p* = 0.887) or the stroke group (*t* (11) = −0.122, *p* = 0.905) (Table 1). HABP (hyaluronic acid binding protein) immunohistochemistry staining showed that HA was localized in IMCT (including endomysium, perimysium, and epimysium) in the forelimb muscles of the mouse (Figures 3B–E), these images are representative and intended to illustrate HA distribution.

**Table 1:** HA and collagen concentration in IMCT of the forelimb muscle in mouse.

Variable	Ctr-L (*n* = 10)	Ctr-R (*n* = 10)	Stroke-L (*n* = 12)	Stroke-R (*n* = 12)	^#^*p*-value Ctr: L vs. R	^#^*p*-value stroke: L vs. R	^$^*p*-value Ctr-L vs. stroke-L	^$^*p*-value Ctr-R vs. stroke-R
μgHA/g muscle	50.07 ± 8.84	49.36 ± 12.22	63.74 ± 11.80	64.16 ± 13.52	0.887	0.905	0.022*	0.015*
μgCol/g muscle	8.62 ± 2.91	8.77 ± 1.35	8.24 ± 2.11	8.28 ± 2.45	0.833	0.956	0.959	0.923

^#^Paired *t*-test, ^$^ANOVA with Dunnett’s post hoc test. Ctr, control; L, left side; R, right side. Data are presented as the mean ± SD. *p < 0.05.

### 3.3 Quantification and distribution of collagen in the forelimb muscles

**Figure 4:**
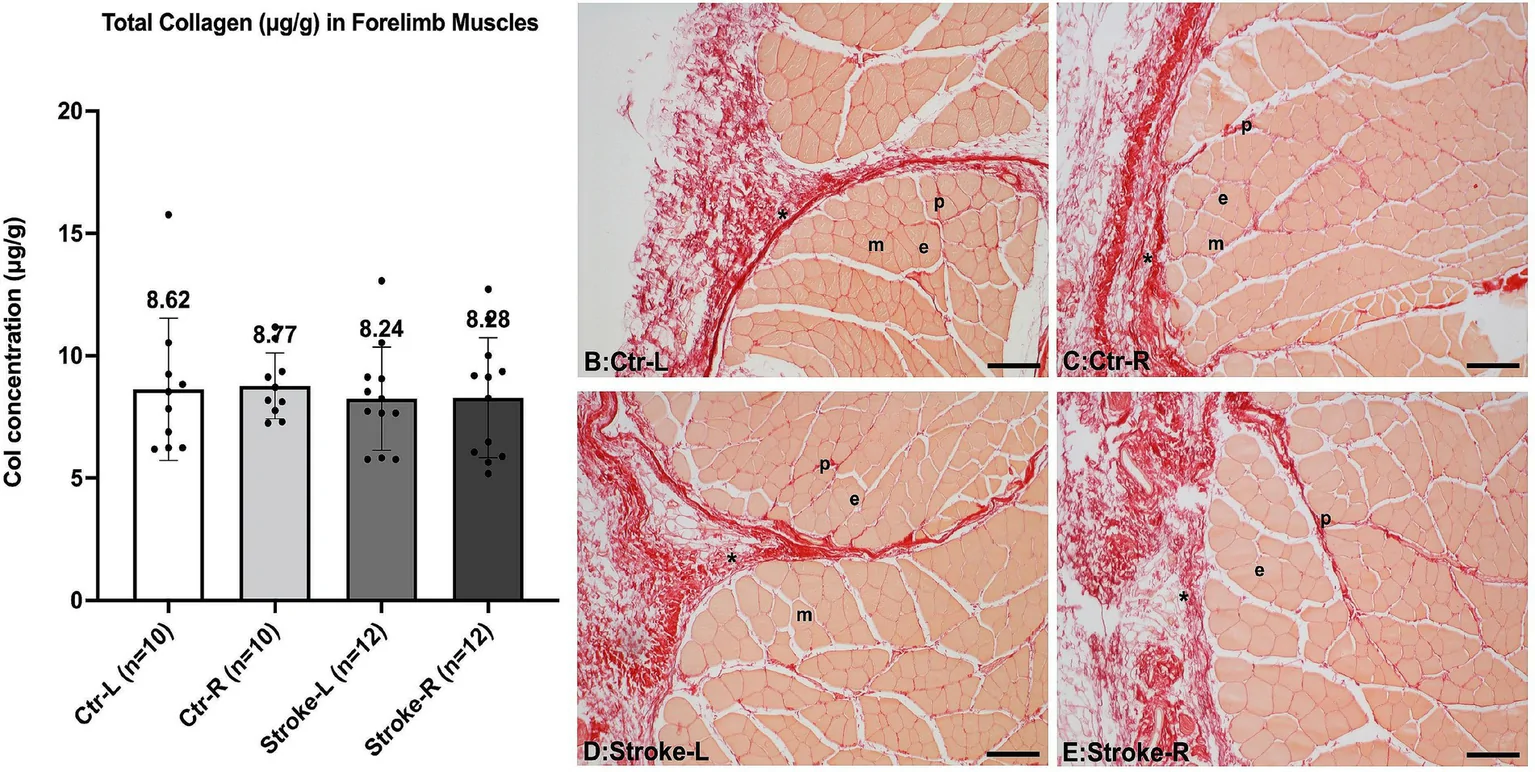
**(A)** Total collagen concentration in forelimb muscles. The collagen concentration in the left and right forelimb muscles on the left (Ctrl-L), the right (Ctrl-R) side of control mouse, and on the left (Stroke-L, stroke affected) side, the right (Stroke-R, non-affected) side of stroke mouse (Data are presented as the mean ± SD, *p* > 0.05). Paired *t*-tests were applied to compare the collagen concentrations between the left and right forelimb muscles within the same animals in the control and experimental group. ANOVA with Dunnett’s post hoc test was applied to compare the Col concentrations between the left and right sides of the experimental group with the left and right sides control group separately. **(B–E)** Collagen distribution **(**Picrosirius Red Staining) in IMCT of the forelimb muscle. **(B)** Control left side; **(C)** control right side; **(D)** stroke left (affected) side; **(E)** stroke right (unaffected) side. *, epimysium; p, perimysium; e, endomysium; m, muscle fiber. Scale bars: 100 μm. Collagen is in red, the muscle fibers of the forelimb muscle in yellow.

The collagen concentrations in the forelimb muscles were: 8.62 ± 2.91 μg/g on the left and 8.77 ± 1.35 μg/g on the right side of the sham surgery mouse in the control group; 8.24 ± 2.11 μg/g on the left (stroke affected) side and 8.28 ± 2.45 μg/g on the right (non-affected) side of the stroke mouse in the experimental group (Figure 4A). The results showed that there was no statistical difference in collagen concentration in the forelimb muscle between the control and experimental groups (left: *p* = 0.959; right: *p* = 0.923). And within group comparisons showed no significant difference of the collagen concentration in the forelimb muscle between the left and right side of the control group (*t* (9) = −0.217, *p* = 0.833) and experimental group (*t* (11) = −0.056, *p* = 0.956) (Table 1). Picrosirius red staining of the forelimb muscle demonstrated the presence and spatial localization of collagen (in red) within the IMCT (including epimysium, endomysium, and perimysium) in the forelimb muscle of the mouse (Figures 4B–E).

## 4 Discussion

Our study investigated the changes in HA and collagen concentrations in the forelimb muscles of mice following post distal middle cerebral artery occlusion (dMCAO) stroke, comparing both the affected and the unaffected sides with those of the sham controls. The main finding of this study is that forelimb muscles from mice examined 4 weeks after dMCAO displayed significantly higher HA concentrations than sham-operated controls, whereas collagen concentrations remained unchanged. These findings indicate that measurable alterations of the intramuscular extracellular matrix occur during the early chronic stage after stroke and suggest that HA remodeling may precede detectable collagen accumulation.

At 4 weeks post right dMCAO-induced stroke, HA concentrations in the forelimb muscles were approximately 25% higher on both the affected and unaffected sides than that in sham controls. HA is a high-molecular-weight glycosaminoglycan (GAG) widely distributed in different tissues, playing a crucial role in maintaining mechanical stability, acting as a water reservoir, and facilitating lubrication (35). Its key mechanical function is to regulate tissue viscosity, enabling smooth gliding between adjacent fascia or intramuscular connective tissue (IMCT) and muscle (24, 31, 36, 37).

**Figure 5:**
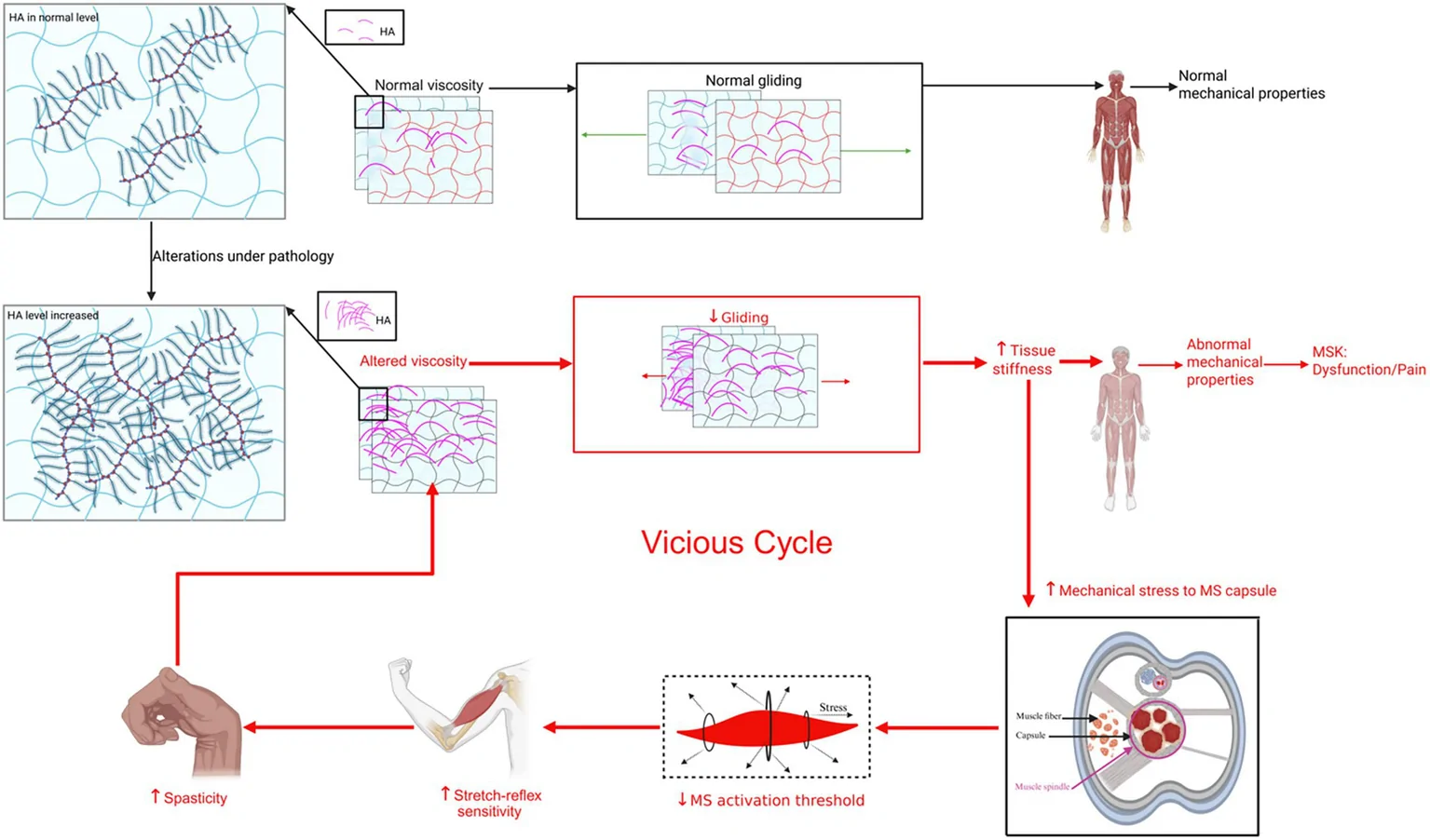
Schematic illustration of proposed relationship between HA alteration and post stroke spasticity.

Our observations align with an MRI study in humans that detected elevated GAG, primarily composed of HA, in the upper limbs of post-stroke patients with spasticity, which decreased after hyaluronidase injection (38). Previous studies have suggested (17, 25, 39), the increase in HA concentration can lower the activation threshold of muscle spindle afferents, which increases reflex excitability that aggravate the stretch reflex that potentially exacerbate post stroke spasticity (Schematically illustrated in Figure 5). Given the established role of HA in regulating tissue lubrication and viscoelastic behavior, the increase in HA concentration observed in this study may reflect alterations in extracellular matrix composition that could influence tissue mechanical properties. However, tissue viscosity, gliding behavior, and stiffness were not directly assessed in the present study. Therefore, whether increased intramuscular HA contributes to clinical manifestations such as spasticity remains to be established through direct experimental investigation.

Moreover, the systemic increase of HA observed in both the stroke affected and unaffected limbs indicates that unilateral stroke can trigger HA adaptations even in the unaffected limb. The HA alterations on unaffected side can change the tissue viscosity which limits muscle fiber and IMCT gliding, which potentially generate musculoskeletal (MSK) disorders such as pain or dysfunctions (24, 36, 40). This suggests a broader ECM adaptation beyond the site affected by central neural pathology associated with stroke, which are more global than previously understood, implying that post-stroke symptoms in the musculoskeletal (MSK) system may be more widespread than traditionally recognized.

The unchanged collagen level is consistent with an animal study in the subacute stage of stroke (41). This study compared collagen alterations across immobilization alone, stroke alone, and stroke combined with immobilization. Collagen density was evaluated semi-quantitatively using Picrosirius Red staining and image analysis. The study reported no significant change in collagen density within endomysium and perimysium in forelimb muscle in subacute stroke rats, whereas collagen increased in the immobilization group and the stroke with immobilization group. These findings suggest that immobilization leads to more pronounced collagen accumulation in IMCT than stroke alone (41). In our study, the absence of significant collagen remodeling may be attributed to the relatively mild motor deficits observed, as indicated by the gridwalk test. Such mild impairments may not cause sufficient mechanical stress to activate fibroblasts and initiate collagen deposition processes that are more commonly associated with more severe or chronic motor dysfunction (42).

In chronic stroke, an ultrasound study in humans reported a significant increase in fascia thickness in lower limb deep fascia on the stroke affected side in chronic stroke patient with spasticity (43). The increased deep fascia thickness may reflect enhanced collagen fiber organization or increased collagen content (44). However, these studies differ in assessment methods, type of subjects, stroke stage, and the presence of secondary conditions such as spasticity and immobilization. Therefore, direct comparisons of their findings are limited. From a temporal perspective, collagen levels appear to remain unchanged during the subacute and early chronic stages, while increased fascia thickness is observed in the chronic stage. This pattern suggests that post-stroke collagen alterations may emerge gradually and develop over a longer timeframe. The earlier accumulation of HA, in comparison to collagen, may reflect its rapid remodeling and sensitivity to mechanical stress (17). This altered tissue viscosity can reduce IMCT gliding to increase stiffness of IMCT and muscle, which may trigger collagen deposition and fibrosis (17). Therefore, a longitudinal study with longer timeframe is needed to investigate the timeline of post stroke collagen remodeling. That will help to further understand the therapeutic window of ECM targeted interventions.

Our finding of systemic hyaluronan accumulation in the forelimb muscles of mice at early chronic stroke stage suggests that the impact of stroke on MSK system is widespread. Targeting extracellular matrix components such as hyaluronan could help reduce spasticity on stroke affected side and prevent muscle stiffness and myofascial pain on the contralateral side. Future clinical interventions (such as hyaluronidase injection (38)) aimed at modulating extracellular matrix composition, might provide therapeutic benefits, and enhance functional recovery for stroke survivors.

### 4.1 Limitations and further research

This study quantified HA concentration but did not evaluate its molecular weight, which is also important for HA’s bioactivity. Although total collagen was assessed, future studies should distinguish collagen subtypes (e.g., type I and type III). In addition, future work should also investigate other ECM components, including elastic fibers. Moreover, the nature of the observational study limits the ability to attribute the ECM changes to a specific causal pathway. Future mechanistic studies using controlled experiments are needed to investigate the roles of interacting factors such as inflammatory, mechanical, and cellular responses in ECM remodeling. Such work would help clarify their specific contributions and interactions. Finally, HA and collagen were assessed only at an early chronic post-stroke time point (4 weeks). Longitudinal sampling across multiple time points is required to define the onset of HA and collagen alteration, thereby clarifying the temporal dynamics of ECM remodeling, and identifying therapeutic windows for ECM-targeted interventions.

## 5 Conclusion

This study demonstrates an increase in HA, while collagen levels remained unchanged, in forelimb muscles at 4 weeks following right dMCAO-induced stroke in mice, providing biochemical evidence of alterations in ECM composition during the early chronic stage after stroke. The observed bilateral increase in HA suggests that ECM alterations may not be restricted to the limb contralateral to the cerebral lesion. Whether these biochemical changes translate into functional or clinical consequences requires further investigation. Longitudinal studies are needed to define the temporal progression of ECM changes and to identify potential therapeutic windows.

## Data Availability

The original contributions presented in the study are included in the article/supplementary material, further inquiries can be directed to the corresponding author.
